# Modeling a superficial radiotherapy X‐ray source for relative dose calculations

**DOI:** 10.1120/jacmp.v16i3.5162

**Published:** 2015-05-08

**Authors:** Christopher D. Johnstone, Richard LaFontaine, Yannick Poirier, Mauro Tambasco

**Affiliations:** ^1^ Department of Physics San Diego State University San Diego San Diego CA USA; ^2^ Department of Radiation Oncology Naval Medical Center San Diego San Diego CA USA; ^3^ Department of Medical Physics CancerCare Manitoba Winnipeg MB Canada; ^4^ Department of Physics & Astronomy University of Calgary & Tom Baker Cancer Centre Calgary AB Canada; ^5^ Department of Oncology University of Calgary & Tom Baker Cancer Centre Calgary AB Canada

**Keywords:** superficial radiotherapy, absorbed dose computation, X‐ray source, PDD, profiles

## Abstract

The purpose of this study was to empirically characterize and validate a kilovoltage (kV) X‐ray beam source model of a superficial X‐ray unit for relative dose calculations in water and assess the accuracy of the British Journal of Radiology Supplement 25 (BJR 25) percentage depth dose (PDD) data. We measured central axis PDDs and dose profiles using an Xstrahl 150 X‐ray system. We also compared the measured and calculated PDDs to those in the BJR 25. The Xstrahl source was modeled as an effective point source with varying spatial fluence and spectra. In‐air ionization chamber measurements were made along the x‐ and y‐axes of the X‐ray beam to derive the spatial fluence and half‐value layer (HVL) measurements were made to derive the spatially varying spectra. This beam characterization and resulting source model was used as input for our in‐house dose calculation software (kVDoseCalc) to compute radiation dose at points of interest (POIs). The PDDs and dose profiles were measured using 2, 5, and 15 cm cone sizes at 80, 120, 140, and 150 kVp energies in a scanning water phantom using IBA Farmer‐type ionization chambers of volumes 0.65 and 0.13 cc, respectively. The percent difference in the computed PDDs compared with our measurements range from −4.8% to 4.8%, with an overall mean percent difference and standard deviation of 1.5% and 0.7%, respectively. The percent difference between our PDD measurements and those from BJR 25 range from −14.0% to 15.7%, with an overall mean percent difference and standard deviation of 4.9% and 2.1%, respectively — showing that the measurements are in much better agreement with kVDoseCalc than BJR 25. The range in percent difference between kVDoseCalc and measurement for profiles was −5.9% to 5.9%, with an overall mean percent difference and standard deviation of 1.4% and 1.4%, respectively. The results demonstrate that our empirically based X‐ray source modeling approach for superficial X‐ray therapy can be used to accurately compute relative dose in a homogeneous water‐equivalent medium. They also show limitations in the accuracy of the BJR 25 PDD data.

PACS number: 87.55.kh

## I. INTRODUCTION

Superficial radiation therapy utilizes kilovoltage (kV) X‐rays to treat various benign and malignant skin lesions on or near the surface of the skin (i.e., within 3 mm of the surface). It is therefore ideal for treating superficial lesions such as basal and squamous cell carcinoma, keloid scars, mycosis fungoides, psoriasis, benign plaques, and other dermatological conditions.^1^, ^2^

Total dose for superficial therapy treatments can be as high as 45 Gy (4.5 Gy/fraction) to 85 Gy, given in daily fractions ranging from 8 to 17 fractions.^3^, ^4^, ^5^ For unique cases of basal cell carcinomas with lesions sizes less than 3 cm,^(6)^ treatment composed of a single fraction of up to 18 Gy may be given.^(7)^ These dose fractionation treatments are large enough to cause an acute radiotherapy reaction, possibly leading to erythema of the skin characterized by desquamation, oozing, crusting of the lesion, skin depigmentation, skin atrophy, telangiecasia, and hair loss.^(3)^ In some cases, dose to underlying normal tissue may go beyond their radiation tolerances, which can lead to an increased risk of secondary cancers and the development of normal tissue complications due to deterministic effects.^(8)^ Hence, there is a need for a clinically feasible method to accurately assess patient dose to ensure that the target area receives the intended dose and the dose to normal tissues does not exceed their radiation tolerances. In order to do so, it is necessary to have an accurate model of the X‐ray source.

In this study, we use an in‐house kV X‐ray dose computation system (kVDoseCalc)^(9)^ to investigate a technique to empirically characterize and validate a kV X‐ray beam source model of a superficial radiotherapy unit through measurements that can be easily performed with standard equipment available in a clinical medical physics facility. The validation is done by comparing experimental percentage depth dose (PDD) and profile dose measurements to dose computations performed using the empirically derived X‐ray source data as input for kVDoseCalc. Our X‐ray source model may be coupled with other superficial dose computation software, such as those created by Bartzsch and Oelfke^(10)^ and Verhaegen et al.^(11)^ Additionally, the PDD results of our dose computations are compared to PDDs published in the British Journal of Radiology Supplement 25 (BJR 25),^(12)^ where the PDDs from the BJR 25 generally serve as a comparison to experimentally measured PDDs of superficial X‐ray therapy.^13^, ^14^, ^15^ This study also demonstrates the limitations of the BJR 25 PDD data.

## II. MATERIALS AND METHODS

### A. Equipment

**Figure 1:**
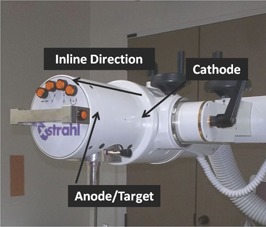
The Xstrahl 150 unit with the inline direction, cathode, and anode/target labeled.

The superficial X‐ray therapy unit used in this study is an Xstrahl 150 X‐ray system (Gulmay Medical Ltd., Surrey, United Kingdom) (Fig. 1). The unit has peak tube voltages of 10 to 150 kVp and tube currents of 0 to 30 mA, and a maximum power output of 3 kilowatts. The unit's X‐ray tube specifications include a minimum and maximum HVL of 0.2 mm Al and 1.0 mm Cu (13 mm Al equivalent), respectively, 0.8±0.1 mmBe internal filtration, focal spot size of 7.5 mm, and a tungsten target (30° angle).

The unit contains a range of applicator cones ranging from 1.5 to 15 cm in base diameters. Energies of 80, 120, 140, and 150 kVp were used in this study, and beam characterization measurements using these energies were done using the 2, 5, and 15 cm applicator diameters with source‐to‐surface distances (SSDs) of 15, 15, and 25 cm, respectively, for all PDD and profile measurements. The bottoms of the applicators are in contact with the surface of the water (simulating patient treatments) for all PDD and profile measurements. The added filtrations and central‐axis HVLs at each beam characterization energy are shown in Table 1.

**Table 1:** Added filtration and central‐axis HVLs at each energy. Standard deviations of all central‐axis HVLs within ±0.1 mm Al.

*Energy (kVp)*	*Added Filtration*	*Measured HVL (mm Al)*
80	1.70 mm Al	2.06
120	0.05 mm Cu+0.90 mm Al	4.18
140	0.20 mm Cu+1.15 mm Al	7.14
150	1.00 mm Cu+1.20 mm Al	13.40

All dose measurements in this study were done using an IBA Blue Phantom 2 scanning water phantom and DOSE‐1 electrometer (IBA Dosimetry, Schwarzenbruck, Germany) coupled with OmniPro‐Accept 7 dosimetry software. PDD and HVL measurements for the 5 and 15 cm applicator diameter used the IBA FC65‐G Farmer‐type ionization chamber (0.65 cc) calibrated by the accredited dosimetry calibration laboratory (UWADCL). The IBA CC13 compact ion chamber (0.13 cc) was used for the PDD measurements of the 2 cm applicator diameter, and for the 2, 5, and 15 cm applicator diameter profile measurements. According to the APPM Task Group 61 (AAPM TG‐61),^(16)^ cylindrical type ionization chambers, such as the ones used in this study, have nearly constant energy response in the energy ranges of 40 to 300 kVp, and this was one of the reasons they were chosen for the measurements in this study.

### B. HVL measurements

**Figure 2:**
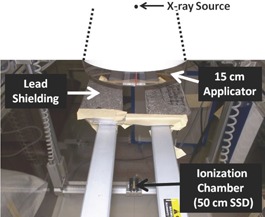
Experimental setup for our central and off‐axis HVL measurements.

Both central and off‐axis HVL measurements were acquired using the AAPM TG‐61 protocol.^(16)^ Due to setup limitations, we were unable to achieve a 50 cm diaphragm‐to‐detector distance as recommended by the AAPM TG‐61, and our field size was not “narrow” (approximately 2 cm by 8 cm). To obtain the HVL from only the primary beam with as little scatter as possible, we placed the chamber in the IBA Blue Phantom 2 tank without water, 50 cm from the source and 21 cm from the bottom of the tank (Fig. 2). We made off‐axis HVL measurements inside the empty water tank to achieve positioning accuracy within ±0.1 mm.^(17)^ Measurements were made off‐axis every 3 cm from the center of the field out to 18 cm for the inline and crossline directions. To shield against scatter, we placed a 3 cm slab thickness of lead at the base of the applicator (Fig. 2). The slab contained a cutout across its length to allow for both central and off‐axis HVL measurements. We measured the output of the open beam, and added millimeter thicknesses of aluminum (Al) until we obtained Al thicknesses that were both below and above the actual HVL. To determine the HVL, we used a three‐point semilogarithmic interpolation technique for both central and off‐axis HVL calculations.^18^, ^19^

### C. PDD measurements

**Figure 3:**
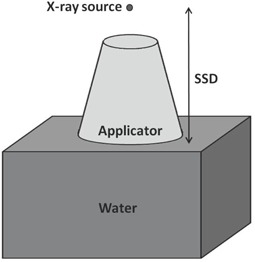
Experimental setup for our PDD and profile dose measurements.

The PDD measurements were taken from 1 to 10 cm depths in 1 cm increments along the central axis of the water phantom, starting at the 10 cm depth, to minimize unwanted water movement that may skew readings. Hill et al.^(20)^ recommends against using an ionization chamber at a depth shallower than the radius of the chamber. Our shallowest depth is 1 cm, which is much larger than our Farmer chamber radius (0.3 cm). PDD measurements were made for the 2, 5, and 15 cm applicator diameters at 80, 120, 140, and 150 kVp, with a 10 mAs tube current and 0.5 s scan time. To simulate actual patient setup, all measurements were taken with the applicators in contact with the water's surface. Figure 3 illustrates the PDD setup (as well as the profile setup). Due to setup limitations, we were unable to position the chambers for surface dose readings. To obtain the surface dose, we used the “polyfit” function in MATLAB (MathWorks, Natick, MA) to perform a polynomial extrapolation of the PDDs to the surface.

### D. Dose computation software

#### D.1 kVDoseCalc

An in‐house kV X‐ray dose computational tool (kVDoseCalc) has been used to validate dose calculations using the beam characterization and corresponding X‐ray source model approach presented in this study.^18^, ^21^ Details on the physics, algorithms, and validation of this in‐house software are given by Kouznetsov and Tambasco^(9)^ and Poirier et al.^(21)^ Briefly, kVDoseCalc uses both a deterministic and stochastic approach to numerically solve the linear Boltzmann transport equation. These calculations take into consideration the primary, first‐scatter, and higher order scatter components of the X‐ray beam and calculate dose at a point of interest (POI) within seconds. Dose is calculated using computed tomography (CT) voxel data imported into kVDoseCalc in the standard digital imaging and communication in medicine (DICOM) format. The software includes photoelectric, coherent, and incoherent Compton scattering interactions, but electron transport is not modeled as it is assumed that their relatively short ranges (maximum of 0.04 mm in water for a 150 kVp beam) are locally absorbed.^(9)^ This dose computation tool has been validated against Electron Gamma Shower National Research Council (EGSnrc, National Research Council of Canada, Ottawa, Canada) and Monte Carlo N‐Particle Transport Code (MNCP, Los Alamos National Laboratory, Los Alamos, USA) in heterogeneous media.^(9)^ Additional validation has also been performed in both homogeneous and heterogeneous media as computed dose compared against experimental measurement.^(21)^

#### D.2 Phantom modeling

MATLAB was used to construct a virtual homogeneous water phantom of size $41.2\times 41.2\times 32.4\;{\text{cm}}^{3}$ in DICOM format. The material composition of the phantom were imported into kVDoseCalc and consisted of 512×512×108 voxels ($28.3\times 10^{6}$ total), each of size $0.081\times 0.081\times 0.30\;{\text{cm}}^{3}$. Hounsfield unit (HU) ranges were mapped to the physical density ranges of air and phantom material (water). Air and water were assigned a material composition by inputting the elements and corresponding nuclear densities of each respective material. The macro cross sections used by kVDoseCalc to compute dose were calculated using the ENDF/B‐VI micro cross section library.^(22)^ In principle, the same process could be carried out on a patient CT dataset. However, this would require appropriate HU‐to‐density and density‐to‐medium characterization, which is currently a subject of ongoing research.^23^, ^24^, ^25^, ^26^

### E. Beam characterization and corresponding source model

Our source model consists of an effective virtual point source located at the same position as the real physical source. Both the primary and scattered radiation is taken to originate from the effective point source to produce a spatially varying fluence and spectral distribution in the plane at base of the applicator at the SSD defined by the applicator sizes of the Xstrahl 150 X‐ray system. Following studies by Poirier et al.,^18^, ^21^ we characterized the beam fluence through relative in‐air dose measurements at the base of the applicators and characterized the spectral aspect of the X‐ray beam from the kVp setting and empirical measurements of HVL. We characterized spatial variations of the beam spectra due to inherent and added filtration and the heel effect along the inline direction (i.e., the anode–cathode tube direction) illustrated in Fig. 1. The spatially varying fluence and spectral distribution are back‐projected from the location of the plane at the base of the applicator to X‐ray source location to construct the effective point source distribution function that models the primary and scattered radiation and associated penumbra effects.

#### E.1 Beam fluence

We used the method developed by Poirier et. al.^(18)^ to model the spectral and spatial fluence of the X‐ray beam. In this method, the beam is defined by a planar fluence differential in energy, which was assumed to be a separable function as follows:

(1) $$\phi (x,y,E)=X(x)Y(y)U(x,E)\phi_{0}$$

where X(x)Y(y) represent the relative photon distribution taken to vary independently in the inline (x) and crossline (y) directions, respectively (Fig. 1). The parameter U(x,E) represents the beam spectrum in which variations were found to be present only in the inline direction of the beam, as this is the direction that exhibits the heel effect. This spectrum is generated using third‐party software Spektr^(27)^ and SpekCalc.^(28)^ The parameter $\theta_{0}$ represents a photon distribution calibration constant relating the dose computed by kVDoseCalc to experimentally measured relative dose.

Following the method by Poirier et al.,^(18)^ we expressed the spatially varying fluence in terms of relative in‐air dose and spectral measurements as follows:

(2) $$\frac{X(x)}{X(0)}=\frac{D^{air}(x,0)\int_{0}^{E_{\text{max}}}U(0,E){\left(\frac{{\bar{\mu }}_{en}}{\rho }\right)}_{air}EdE}{D^{air}(0,0)\int_{0}^{E_{\text{max}}}U(x,E){\left(\frac{{\bar{\mu }}_{en}}{\rho }\right)}_{air}EdE}$$

where ${\bar{\mu }}_{en}/\rho$ is the mass energy‐absorption coefficient, and the absorbed dose in‐air is given by

(3) $$D^{air}(x,y)=\int_{0}^{E_{\text{max}}}\phi (x,y,E){\left(\frac{{\bar{\mu }}_{en}}{\rho }\right)}_{air}EdE.$$

Calculations in the Y direction are performed in the same fashion.

The relative in‐air dose measurements were made along the crossline and inline directions at the base of the applicators using the CC13 compact ionization chamber. Measurements were made every 0.25 cm for the 2 cm applicator and every 0.5 cm for the 5 and 15 cm applicators. Deviations of our beam fluence, relative to the center of the beam, were measured with less than a 2% standard deviation within 75% of the center of the beam and up to 10% at the applicator edges. These standard deviations were obtained by using the dose at points throughout the profile of the beam and comparing them to the relative dose at the very center of the beam.

These cross‐sectional spatial intensities exiting the base of the applicators were used to model the varying beam fluence in kVDoseCalc.

Dose computations were performed for a single stationary X‐ray beam placed directly above the water phantom, along its central axis, at SSDs of 15 cm for the 2 and 5 cm field applicators and 25 cm for the 15 cm applicator. In this study, circular field sizes were collimated at 2, 5, and 15 cm diameters and $5\times 10^{5}$ particles were used to compute dose. Computational accuracy in dose varied by 3% using $2.5\times 10^{5}$ particles and less than 1% with the use of $1\times 10^{6}$ particles when compared to $5\times 10^{5}$ particles. Thus, we used $5\times 10^{5}$ particles as a reasonable compromise between speed and accuracy. Based on a four Intel Core 7 960 CPU (Intel Corporation, Santa Clara, CA) at 3.20 GHz, an average time of 24 s is required to compute dose at a POI.^(21)^

#### E.2 X‐ray beam spectra

To generate the spectra, we used two freely available software: Spektr by Siewersden et al.,^(27)^ that utilizes Boone and Seibert's spectral model^(29)^ and SpekCalc developed by Poludniowski et al.^(28)^ Both software calculate X‐ray spectra using values for kVp, and internal and external filtration input by the user. In this work, nominal values were used for the kVp and external filtration. The internal filtration was tweaked using an iterative approach to obtain a best match between the HVL of the generated spectra and measured HVL values. We measured HVL in both the crossline and inline directions of the X‐ray tube using the method described in the Materials & Methods section B. The measurements in the crossline direction were performed to verify that HVL (and therefore spectra) did not vary along this direction. Values for the spectrum were calculated at various points along the inline direction to derive spatial variations of the fluence according to Eq. (3), as explained in the Materials & Methods section E.1

We used SpekCalc for the 80 and 150 kVp beams and Spektr for 120 and 140 kVp beams (our measured HVLs were beyond the range for Spektr to generate spectra at 80 and 150 kVp). It is worth noting that dose calculations generated using Spektr and SpekCalc for equivalent HVL and kVp combinations compared to within a 1% difference of each other.

The 2 cm applicator used in this study was small enough to assume a uniform spectral distribution at the base of the applicators without compromising the computational accuracy of kVDoseCalc simulation for all energies; however, the spatial distribution of fluence was still measured. This uniform spectral assumption held true for the 5 cm applicator as well, with the exception of the inline direction at 140 and 150 kVp. The 15 cm applicator was too large to make this assumption, and the varying spectrum at the base of the applicator was accounted for by measuring the off‐axis HVL by applying the method in described in the Materials & Methods section B. Spatial beam intensities at the base of all three applicators (2, 5, and 15 cm applicator diameters) were also measured.

### F. Comparison of measured and BJR 25 PDDs

The low‐energy PDDs contained within the BJR 25 are only averages, as they were compiled over eight institutions and averaged together using different superficial radiotherapy machines. Low‐energy PDD data of the BJR 25 was acquired from the BJR 17, where accuracy discrepancies may be due to the use of compiling data from interpolated HVLs and multiple types of phantom materials (water, Mix‐D, Temex, Scanplas, tissue‐equivalent plastic, and polystyrene).^(30)^ Different phantom materials, such as polystyrene, can have a percent difference as high as 17.6% when compared to water.^(31)^ In this study, we compared our experimentally measured PDDs to the PDDs of the BJR 25. In this comparison, beam quality (HVL) and technique settings (kVp, SSD, and applicator size) were used, comparing against the exact kVp, SSD, and applicator sizes of the BJR 25, and to the nearest match of HVL.

### G. Dose profiles

Profile measurements were made to validate the accuracy of the X‐ray source model for off‐axis dose calculations. All profile measurements were setup in the same way as the PDD measurements, but were taken at a constant depth of 1 cm. The CC13 ionization chamber was used for all three applicator diameters to achieve better accuracy for the high‐dose gradient regions of profile measurements. Measurements for the 80, 120, 140, and 150 kVp settings were made every 0.5 cm in the relatively flat central regions of the profiles and every 0.1 cm in the high‐dose gradient regions near the ends of the applicator, from −3 to +3, −5 to +5, and −12 to +12 cm in the inline direction for the 2, 5, and 15 cm applicator diameters, respectively. The measurements were normalized to the central‐axis beam output for all profiles.

## III. RESULTS

### A. HVL measurements

Central‐axis HVLs for the kVp settings used are shown in Table 1. The heel effect was found to be present only in the inline direction at 140 and 150 kVp with the larger 15 cm field size. It is only marginally present in the 5 cm applicator at 140 and 150 kVp, and not present in the 2 cm applicator.

The standard deviations for central‐axis and off‐axis HVL measurements are within ±0.1 mm Al and ±0.3 mm Al, respectively, as determined by three measurements at each point and kVp setting, testing the repeatability. For the differences of HVL values between central and off‐axis points, the standard deviations in the inline direction when compared to the center of the beam were as high as ±0.3 mm Al near the edges of the applicators and reduced to ±0.1 mm Al towards the center of the applicators. Similarly, the standard deviation in the crossline direction when compared to the center of the beam were as high as ±0.2 mm Al near the edges of the applicators and reduced to ±0.1 mm Al toward the center of the applicators.

### B. Comparison of measured and computed PDDs

**Figure 4:**
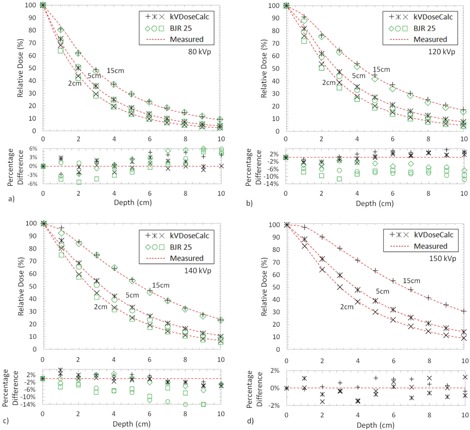
Measured PDDs compared to kVDoseCalc and BJR 25 for the 2, 5, and 15 cm applicator diameters at 15, 15, and 25 cm SSDs, respectfully, and energies (a) 80 kVp, (b) 120 kVp, (c) 140 kVp, and (d) 150 kVp. The SSD and field sizes in the BJR 25 at 150 kVp could not be compared to the SSD and field sizes of our measurements. Percentage difference in dose between kVDoseCalc, BJR 25, and experimental measurements are displayed underneath.

For all of the applicator diameters and kVp settings, the relative PDD data generated from kVDoseCalc coincided with our scanning water phantom measurements within a percent difference range of −4.8% to 4.8%, and with an overall mean percent difference and standard deviation of 1.5% and 0.7%, respectively (Fig. 4). These results show good agreement between measurement and kVDoseCalc. The largest deviations are few (over 90% of points within a 3% difference) and appear to be random at both shallow and deep depths for 80, 120, and 140 kVp, and all differences are within 2% at 150 kVp. The PDDs were taken as the average of three dose readings at each measurement point, with an average reading uncertainty of 0.3% over all measured points. A setup uncertainty of 2.1% was estimated by disassembling the original setup and reassembling it for all measurements with the 5 cm applicator. Adding the setup and dose reading uncertainties in quadrature gives a total dose measurement uncertainty of 2.1%.

### C. Comparison of measured and BJR 25 PDDs

The percent difference between our measured PDDs and those from BJR 25 range from −14.0% to 15.7%, with an overall mean percent difference and standard deviation of 4.9% and 2.1%, respectively. These results are displayed in Figs. 4(a) to 4(c). Our 150 kVp PDDs could not be compared to the BJR 25 as it used different HVL, applicator geometry, SSD, and kVp.

### D. Dose profiles

**Figure 5:**
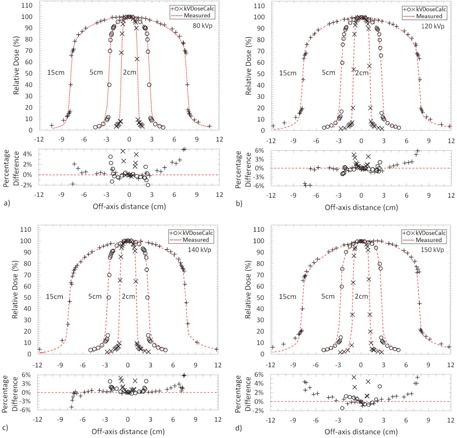
Measured profiles at a 1.0 cm depth compared to kVDoseCalc for the 2, 5, and 15 cm applicator diameters at 15, 15, and 25 cm SSDs, respectfully, and (a) 80 kVp, (b) 120 kVp, (c) 140 kVp, and (d) 150 kVp. Percentage difference in dose between kVDoseCalc and experimental measurements are displayed underneath.

The percent difference in the inline profile data between kVDoseCalc and measurement for all energies ranged from −5.9% to 5.9%, with an overall mean percent difference and standard deviation of 1.4% and 1.4%, respectively, except in the high‐dose gradient regions beyond the edges of the applicators (Fig. 5). Poorer agreement in these very high‐dose gradient regions is likely due to volume averaging effects in the CC13 ionization chamber.^(32)^ The profiles were taken as the average of three dose readings at each measurement point, with an average reading uncertainty of 0.3% over all measured points. A setup uncertainty of 2.1% was estimated by disassembling the original setup and reassembling it for all measurements with the 5 cm applicator. Adding the setup and dose reading uncertainties in quadrature gives a total dose measurement uncertainty of 2.2%. It is important to note that, although the relative percent difference in doses were greater beyond the field edges, the actual differences in absorbed dose rates were within 5 cGy/min, which were within the experimental uncertainty in this region.

That is, the combined HVL and spatial fluencies modeled at the edges of the applicators yielded relatively high deviations (±0.3 mm Al HVL combined with a 10% beam intensity deviation), affecting output by as much as 7 cGy/min.

## IV. DISCUSSION

The aim of this study was to investigate, through experimental validation, a reasonably accurate approach at empirically modeling a superficial radiotherapy X‐ray source for computing relative dose calculations in water. Our results demonstrate good agreement between our computed PDDs and profiles with those experimentally measured, thus validating the approach.

When compared to measurement, the PDDs and dose profiles that were generated by kVDoseCalc when using our beam characterization and corresponding X‐ray source model had an overall mean percent difference and standard deviation within 1.5%. This agreement demonstrates that our approach of utilizing in‐air dose measurements, HVL, and kVp to define the X‐ray beam fluence and spectra is sufficient for characterizing a superficial radiotherapy X‐ray beam for dose computations. It is also worth noting that previous studies have found that kVp and the first HVL can be used to generate spectra that are sufficiently accurate to compute accurate kV X‐ray dose,^18^, ^21^, ^33^, ^34^ and the incorporation of a second HVL to generate more accurate spectra adds no statistically observable improvement to the dose results.^(35)^

The PDDs that kVDoseCalc computes using our beam characterization and corresponding X‐ray source model agree better with measurement than the BJR 25 data for all energies, displayed in Figs. 4(a) to 4(c). The authors suspect that the discrepancy between the measured and BJR 25 PDDs are likely due to the fact that the low‐energy PDD data from the BJR 25 were compiled and averaged over eight separate institutions, using only approximate HVL and kVp with four varying phantom materials.^12^, ^30^ Furthermore, the compiled data provided by BJR 25 are not machine‐specific (composed of varying internal and external filtrations, which can change the spectrum of the beam even for the same HVL and kVp), whereas the results computed with kVDoseCalc were attained from modeling the X‐ray source of the specific machine used to make the measurements. The BJR 25 states that its remeasurement comparisons may be off by as much as 20%, and within 10% with its best agreement.^(12)^ In addition, the BJR 25 acknowledges that the PDD data given in its tables are average values for the reference HVLs, and it also stresses the need for direct measurement if higher accuracy is needed.^(12)^

In a previous study, Jurado et al.^(13)^ discussed the difficulties in directly comparing their PDD curves with those of the BJR 25, stating that their beam filters were compared, but only after linearly interpolating the datasets in BJR 25 to coincide with the specific beam quality and technique settings they used. After interpolation, their PDD comparison was within a 5% difference of the BJR 25 data. A similar study by Evans et al.^(14)^ obtained a percentage difference within 6.2% and, in a study by Hill et al.,^(36)^ the percentage difference was up to 12%. In a study focusing on measuring output factors in a kilovoltage therapy unit,^(33)^ a percentage difference within 5% of the BJR 25's PDD data was obtained at one technique for four different HVL beams.

Although PDD and profile measurements for superficial units have been performed in other studies,^13^, ^14^, ^15^ it is difficult to make a meaningful comparison of the PDD, profile, and reference dose measurements from these previous studies to our study, as the therapy units considered in these previous studies had different inherent and external filtration and operated at different mAs and scanning times. Additionally, there were few techniques that matched the kVp, HVL, SSD, and applicator sizes of this study.

In a study by Jurado et al.,^(13)^ a Pantak Therapax SXT 150 was used, with the “Filter 4” (80 kVp, 2.27 mm Al HVL) being the only technique comparable to the PDD measurements of this study. The “Filter 4” PDDs for their 2, 5, and 15 cm applicators was comparable to our technique of 80 kVp and 2.06 mm Al HVL for the same applicator diameters and SSDs. The percentage difference between the PPDs computed by kVDoseCalc and those measured in the Jurado study were within 2.0%.

## V. CONCLUSIONS

The current clinical practice of superficial radiotherapy does not accurately account for patient contours and tissue heterogeneities. Hence, there is a need to develop an accurate patient‐specific dose computation system for superficial X‐ray therapy procedures. As a first step to this end, it is necessary to have a clinically feasible method to characterize the X‐ray beam and corresponding X‐ray source model for accurate kV dose computations.

This study demonstrates that an empirically based X‐ray beam characterization and corresponding effective point source modeling approach can be used to compute relative dose for superficial X‐ray therapy in water. The validation was shown through our relative central‐axis PDD measurements in water for three different applicator diameters (2, 5, and 15 cm) at four different energies (80, 120, 140, and 150 kVp). The central‐axis PDD and dose profiles computed with kVDoseCalc generally agree within measurement uncertainty. Compared to the central‐axis PDDs measured in the BJR 25, the beam characterization approach used to derive the corresponding effective X‐ray point source for the dose calculations performed with kVDoseCalc yielded results that are approximately twice as accurate, with respect to experimental measurement, than the BJR 25, demonstrating the accuracy limitations of the BJR 25 PDD data. Future work will focus on further experimental validation using anthropomorphic phantoms.

## ACKNOWLEDGMENTS

We wish to thank Lt. Larry Burns and Dr. Theodore St. John for their valuable help and discussion, and Dr. Usha Sinha for acquiring a travel grant (Skolil Fund – Physics Scholarship) for one of the authors (CJ) to carry out part of this study at the Tom Baker Cancer Centre in Calgary, AB, Canada.

## Supporting information

Supplementary Material
