# Supplementary material for: Modeling a superficial radiotherapy X‐ray source for relative dose calculations
Source: J Appl Clin Med Phys. 2015 May 8;16(3):118–30. doi: 10.1120/jacmp.v16i3.5162 (PMC5690109; doi:10.1120/jacmp.v16i3.5162)
Supplement: Supplementary file 1 — Supplementary Material [file ACM2-16-118-s001.docx]

Modeling a superficial radiotherapy x-ray source for dose calculations

Christopher Daniel Johnstone^1^

**Richard LaFontaine^2^**

**Yannick Poirier^3^**

***Mauro Tambasco^1,4,5^**

*Department of Physics^1^
San Diego State University San Diego, California 92182-1233 USA*

*Department of Radiation Oncology^2^
Naval Medical Center San Diego, San Diego, California, 92134-5014 USA*

*Department of Medical Physics^3^
CancerCare Manitoba, Winnipeg, Manitoba, R3E 0V9 Canada*

*Department of Physics & Astronomy^4^
Department of Oncology^5^*

*University of Calgary & Tom Baker Cancer Centre, Calgary, Alberta, T2N 4N2 Canada*

***Corresponding Author:**

Mauro Tambasco, Ph.D., MCCPM

Assistant Professor

Department of Physics

San Diego State University

5500 Campanile Dr.

San Diego, CA, 92182-1233 USA

E-mail: [mtambasco@mail.sdsu.edu](mailto:mtambasco@mail.sdsu.edu)

Phone: 1-[(619) 594-6161](tel:%28619%29%20594-6161)
